# Honey as a Source of Environmental DNA for the Detection and Monitoring of Honey Bee Pathogens and Parasites

**DOI:** 10.3390/vetsci7030113

**Published:** 2020-08-15

**Authors:** Anisa Ribani, Valerio Joe Utzeri, Valeria Taurisano, Luca Fontanesi

**Affiliations:** 1 Department of Agricultural and Food Sciences, University of Bologna, Viale Giuseppe Fanin 46, 40127 Bologna, Italy; anisa.ribani2@unibo.it (A.R.); valeriojoe.utzeri2@unibo.it (V.J.U.); valeria.taurisano2@unibo.it (V.T.); 2 GRIFFA s.r.l., Viale Giuseppe Fanin 48, 40127 Bologna, Italy

**Keywords:** *Apis mellifera*, DNA analysis, epidemiology, health, *Lotmaria passim*, *Melissococcus plutonius*, *Nosema ceranae*, pathology, *Tropilaelaps*, *Varroa destructor*

## Abstract

Environmental DNA (eDNA) has been proposed as a powerful tool to detect and monitor cryptic, elusive, or invasive organisms. We recently demonstrated that honey constitutes an easily accessible source of eDNA. In this study, we extracted DNA from 102 honey samples (74 from Italy and 28 from 17 other countries of all continents) and tested the presence of DNA of nine honey bee pathogens and parasites (*Paenibacillus larvae*, *Melissococcus plutonius*, *Nosema apis*, *Nosema ceranae*, *Ascosphaera apis,* *Lotmaria passim*, *Acarapis woodi*, *Varroa destructor,* and *Tropilaelaps* spp.) using qualitative PCR assays. All honey samples contained DNA from *V. destructor*, confirming the widespread diffusion of this mite. None of the samples gave positive amplifications for *N. apis*, *A. woodi,* and *Tropilaelaps* spp. *M. plutonius* was detected in 87% of the samples, whereas the other pathogens were detected in 43% to 57% of all samples. The frequency of Italian samples positive for *P. larvae* was significantly lower (49%) than in all other countries (79%). The co-occurrence of positive samples for *L. passim* and *A. apis* with *N. ceranae* was significant. This study demonstrated that honey eDNA can be useful to establish monitoring tools to evaluate the sanitary status of honey bee populations.

## 1. Introduction

Environmental DNA (eDNA), defined as DNA extracted from environmental- or organismal-related specimens or matrixes, has been proposed as a powerful tool to detect and monitor cryptic, elusive, or invasive organisms, including parasites and many other pathogens that might be difficult to sample or to identify—also extending or facilitating the possibility to evaluate their distribution, even over time and geographic areas [1,2]. Environmental DNA approaches vary according to the objective, the organisms and, in turn, the methods of specimen collection, DNA isolation, or enrichment for targeted or metagenomics analyses [3,4,5]. The use of eDNA has been recently proposed in the apiculture sector for several purposes [6,7].

The problems determined by the global collapse of honey bee colonies, caused by the co-occurrence of debilitating factors, including environmental stressors and the increased frequency and intensity of chronic diseases, has raised the attention on disease monitoring approaches and pathogen identification methods [8,9]. Prognostic eDNA in the honey bee production systems is detectable in the debris collected from the bottom of the beehive. This material has been proposed as the source of simple and non-invasive collectable specimens useful to detect pathogens and assess the health status of honey bee colonies [10,11,12,13].

Honey constitutes another easily accessible source of eDNA useful for pathogen detection and honey bee epidemiological studies. For example, several authors proposed polymerase chain reaction (PCR) based-assays to identify the presence of *Paenibacillus larvae* and *Melissococcus plutonius* in naturally infected honey samples [14,15,16,17]. These are the two major infecting bacteria in honey bee colonies causing the American and the European foulbroods, respectively [18,19].

Giersch et al. [20] detected the presence of DNA of two microsporidia, *Nosema apis* and *N. ceranae*, in honey sampled in Australia and suggested that the later species was introduced in this continent through either contaminated honey or pollen. These two microsporidia have been significantly associated with the occurrence of colony losses [21,22,23,24,25,26,27,28].

*Ascosphaera apis* is a widespread fungal pathogen of honey bees causing the chalkbrood disease [29] that is responsible for colony losses, mainly when combined with other stressing conditions, derived by the co-presence of microsporidia or mite parasites and derived viruses [30].

Other parasitic microorganisms associated with increased colony mortalities in combination with other health problems and pathogens are two species of trypanosomatids, *Lotmaria passim* and *Crithidia mellificae*, which have been frequently confused and can be distinguished only using molecular methods [31,32,33,34].

Among the honey bee parasites, the tracheal tarsonemid mite *Acarapis woodi* (an internal parasite of the bee tracheal system, distributed in almost all continents; [35]) can causes significant economic damages in some conditions, even if it is not usually considered a real problem despite its quite high prevalence in apiaries of some countries [36].

The ectoparasite mite *Varroa destructor* is far more damaging than the tracheal mite [35]. Varroasis is acknowledged as the worst pest for *Apis mellifera* colonies all over the world, and one of the main responsible for death and reduced populations of overwintering colonies, and a potential factor of colony losses [37,38]. In addition to this mite species, other parasitic mites of the genus *Tropilaelaps* (parasites of *A. dorsata*) have recently raised concern after they have expanded their natural hosts to include *A. mellifera*, after this bee was introduced to Asia [39]. Invasion of *Tropilaelaps* mites in Europe and United States would likely be very damaging for the western apiculture, considering the higher reproduction rate and shorter life cycle that these parasites have than *V. destructor* [40]. To face this emerging threat, monitoring and detection systems have been developed to prevent the introduction of *Tropilaelaps* mites in areas outside Asia, the natural range of these species [41,42].

In this study, we used honey of different botanical and geographical origins as a source of eDNA to detect some of the most important pathogens and parasites of honey bees. We tested and developed qualitative PCR methods to identify two bacteria, *P. larvae* and *M. plutonius*, two microsporidia, *N. apis* and *N. ceranae*, one fungus, *A. apis*, one trypanosomatidae, *L. passim*, and three arthropod parasites, *A. woodi*, *V. destructor* and *Tropilaelaps* spp. The methods applied in this study expanded the possibility to evaluate the occurrence and prevalence of honey bee infecting and parasitizing agents by investigating an unconventional specimen (i.e., honey) than can be useful for epidemiological analyses and monitoring purposes.

## 2. Materials and Methods

### 2.1. Honey Samples

Honey samples were purchased from trade markets or were directly provided by beekeepers. These samples were not from a single hive as they derived by the routine procedures for their preparations and packaging into the final commercialized container or bin. A total of 102 honey samples were collected from European countries (n. 87), North, Central, and South American countries (n. 8), Asian countries (n. 5), African, and Oceanian countries (n. 1 for each continent). The European honey samples were from eight countries (n. 3 from Croatia, n. 4 from Finland, n. 2 from France, n. 1 from Greece, n. 1 from Hungary, n. 74 from Italy, n. 1 from Serbia, n. 1 from The Netherlands). The honey samples from the American regions were from USA (n. 3), Mexico (n. 1), Guatemala (n. 1), Brazil (n. 2), and Chile (n. 1). Asian samples were from China (n. 3), India (n. 1), and Japan (n. 1). The African sample was from Ethiopia and the Oceanian sample was from New Zealand. The year of production of the analysed honey samples ranged from 2004 to 2018 (Figure S1). For 13 samples, the precise year of production was not declared, but according to the collected information, it might be between 2015 and 2017. A total of 60 samples had monofloral and 32 had polyfloral origin, whereas 10 were honeydew honey samples. Table S1 reports the list of samples, including their origin and the year of production.

### 2.2. DNA Extraction from Honey Samples

DNA was extracted from each honey following the protocol previously described [43]. Briefly, for each sample, a total of 50 g of honey was divided into four 50 mL tubes (12.5 g for each tube) then 37.5 mL of ultrapure water was added to each tube. These tubes were vortexed and then incubated at 40 °C for 30 min. In the subsequent step, the tubes were centrifuged for 25 min at 5000× *g* at room temperature and the supernatant was discarded. The obtained pellet was resuspended in 5 mL of ultrapure water and the content of the four tubes was merged in one and then diluted again with ultrapure water. After another centrifugation step (25 min at 5000× *g* at room temperature), the supernatant was discarded and the pellet was resuspended in 0.5 mL of ultrapure water. Then, 1 mL of CTAB extraction buffer [2% (w/v) cetyltrimethylammonium bromide; 1.4 M NaCl; 100 mM Tris-HCl; 20 mM EDTA; pH 8] and 5 μL of RNase A solution (10 mg/mL) were added to each honey pre-treated sample and incubated for 10 min at 60 °C. After the incubation, 30 μL of proteinase K (20 mg/mL) were added and the mix was incubated at 65 °C for 90 min with gentle mixing. Cooled samples at room temperature were centrifuged for 10 min at 16,000× *g*. After the centrifugation, 700 μL of the supernatant was transferred in a tube containing 500 μL of chloroform/isoamyl alcohol (24:1) and mixed by vortexing. This step was followed by a centrifugation for 15 min at 16,000× *g* at room temperature. The supernatant was transferred in a new 1.5 mL tube and the DNA was precipitated with 500 μL of isopropanol and washed with 500 μL of ethanol 70%. Finally, pellets were rehydrated with 30 μL of sterile water and stored at −20 °C until PCR analyses.

Extracted DNA was quality checked using the NanoPhotometer IMPLEN P300 (Implen GmbH, Munchen, Germany) and visually evaluated by 1% agarose gel electrophoresis in TBE 1X buffer, after staining with 1X GelRed Nucleic Acid Gel Stain (Biotium Inc., Hayward, CA, USA).

### 2.3. PCR Analyses

PCR analyses were carried out using the PCR primer pairs reported in Table 1. To assess the possibility to successfully amplify DNA fragments from the extracted DNA, we first verified if amplification could occur for honey bee DNA using primers designed on the mitochondrial DNA (mtDNA) region [44]. Primer pairs used in this study that targeted sequences of *Paenibacillus larvae* (two primer pairs) *Melissococcus plutonius* (two primer pairs) *Nosema apis* (one primer pair), *N. ceranae* (one primer pair), *Ascosphaera apis* (one primer pair), *Lotmaria passim* (one primer pair), *Acarapis woodi* (two primer pairs), and *Varroa destructor* (two primer pairs) were described by other authors (Table 1). Primers to detect the presence of *Tropilaelaps* spp. were designed in this work on the mtDNA sequence of *Tropilaelaps clareae* to target a region of the mitochondrial DNA Cox1 gene that, according to the available sequence information in GenBank, was conserved only in the *Tropilaelaps* genus (Table 1). All selected primer pairs were expected to amplify DNA fragments of <300 bp as the highly degraded honey DNA limits the possibility to successful amplify larger fragments [45].

PCR analyses used one primer pair for each reaction (single PCR analyses). PCR were performed on a 2700 Thermal Cycler (Life Technologies, Waltham, MA, USA) in a total volume of 20 μL including 50 ng of isolated DNA and using KAPA HiFi HotStart Mastermix (Kapa Biosystems, Wilmington, MA, USA) with 10 pmol of each primer and adopting the following PCR profile: initial denaturation step at 95 °C for 3 min, then 35 cycles of alternate temperatures (20 s at 98 °C, 15 s at the specific annealing temperature for the different primer pairs as indicated in Table 1, 30 s at 72 °C), followed by a final extension step at 72 °C for 1 min. Amplified DNA fragments were electrophoresed in 2.5% agarose gels in TBE 1X buffer and stained with 1X GelRed Nucleic Acid Gel Stain (Biotium Inc., Hayward, CA, USA). PCR was carried out at least twice for each primer pair/honey sample combination to confirm the results. Results of these PCR analyses are qualitative (presence or absence of amplification based on the expected DNA fragments).

### 2.4. Sequencing and Data Analysis

Three to seven PCR fragments obtained for each primer pairs from the respective positively amplified honey samples were sequenced. Amplified fragments (derived from 7 μL of PCR product) were purified with 1 μL of ExoSAP-IT^®^ (USB Corporation, Cleveland, OH, USA) for 15 min at 37 °C and then sequenced using the BrightDye^®^ Terminator Cycle Sequencing Kit (NIMAGEN, Nijmegen, The Netherlands). Sequencing reactions were purified using EDTA 0.125 M, Ethanol 100% and Ethanol 70%, following a standard protocol, and then were loaded on an ABI3100 Avant Genetic Analyzer sequencer (Life Technologies) for the detection of DNA sequences. Obtained electropherograms were visually inspected and analysed using MEGA 7 [51] and BioEdit Sequence Alignment Editor v7.0.5 [52] software. BLASTN (http://www.ncbi.nlm.nih.gov/BLAST/) was used to compare and validate the attribution of the obtained DNA sequences to the expected amplified region and organism.

A two-tailed Fisher exact test was used to compare the frequency of positive and negative honey samples between countries or groups of countries. Means were compared using t-Student tests.

## 3. Results

DNA extracted from all honey samples was amplified for the *Apis mellifera* mtDNA targeted region and the obtained fragments were the expected ones [44]. That means that for all samples, DNA was successfully isolated and PCR amplification was not inhibited by any contaminants eventually left in the extracted DNA aliquots (spectrophotometer analyses confirmed this result: A_260_/A_280_ was always >1.6). Therefore, the DNA isolated from all honey samples was then amplified with the other 13 primer pairs in single PCR analysis to obtain information on the presence or absence of nine honey bee pathogens or parasites (*Paenibacillus larvae*, *Melissococcus plutonius*, *Nosema apis*, *N. ceranae*, *Ascosphaera apis*, *Lotmaria passim*, *Acarapis woodi*, *Varroa destructor,* and *Tropilaelaps* spp.). All sequenced fragments for the positively amplified honey samples corresponded to the targeted regions, confirming the specificity of the assays that were developed by other authors (Table 1).

Among the primers that produced positive results, the two primer pairs tested for *P. larvae* gave the same results (presence of amplification or absence of amplification) for 93% of the analysed samples, whereas the two primer pairs used to detect *M. plutonius* gave the same results for 95% of the investigated samples. As the final aim of this study was to obtain information on the presence of potential honey bee health threats, for these two pathogens, we then considered positive a honey sample if at least one or both primer pairs gave a positive amplification.

The two primer pairs tested for *V. destructor* and *A. woodi* were concordant in 100% of the analysed samples, but with opposite results. In the case of *V. destructors,* all 102 honey samples gave positive amplification, whereas in the case of *A. woodi* none of the 102 samples had any amplified fragment. Two other tested PCR assays, each designed for a different organism, did not evidence the presence of the targeted DNA in the extracted honey: *N. apis* and *Tropilaelaps* spp. were never amplified in all honey samples.

**Figure 1:**
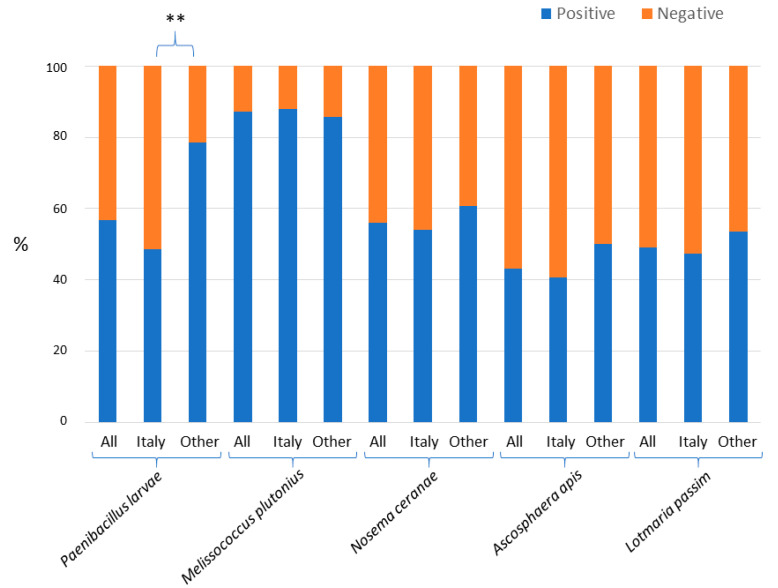
Frequency of the positive (presence of an amplified fragment, in blue) and negative (absence of an amplified fragment, in red) honey samples in the amplifications that targeted *Paenibacillus larvae*, *Melissococcus plutonius*, *Nosema ceranae*, *Ascosphaera apis,* and *Lotmaria passim*. “All” means that all 102 analysed samples were considered together; “Italy” means that only the 74 Italian samples were considered; “Other” means that only the remaining 28 non-Italian samples were considered. The two asterisks (**) mean that the difference of positive honey samples between the two contrasted groups was highly significant (*p* < 0.01).

Figure 1 shows the percentage of honey samples that contained DNA of the other pathogens and parasites (i.e., *P. larvae*, *M. plutonius*, *N. ceranae*, *A. apis,* and *L. passim*). This figure reports the results considering all 102 honey samples together or dividing them into two other groups: only Italian samples (n. 74) and samples from all other countries (n. 28). *Melissococcus plutonius* was detected in 87% of the analysed samples whereas the other pathogens were detected in 43% to 57% of all samples. Comparing the results obtained for the Italian samples with the results obtained for all non-Italian samples, significant difference was observed for *P. larvae* (*p* = 0.0074; two tailed Fisher exact test). The frequency of positive samples was significantly lower in Italy (49%) than in all other countries considered all together (79%). This result was also confirmed dividing the non-Italian samples in other two groups: samples from all other European countries (n. 13) and samples from all other continents (n. 15) which both had a higher frequency of positive samples for *P. larvae* (77% with *p* = 0.075 and 80% with *p* = 0.044, respectively) when compared to the Italian samples. For the *M. plutonius*, *N. ceranae*, *A. apis,* and *L. passim* no significant differences between the Italian samples and the non-Italian samples were observed.

Considering the stratification of the results based on north and south of Italy, including the Sicily and Sardinia islands in this later geographic area (n. 30 vs. 31 samples; we excluded the regions of Central Italy for the low number of remaining samples), *M. plutonius* resulted significantly less frequent in samples from the north of Italy (*p* = 0.0125) whereas *L. passim* resulted more frequent in samples from this part of Italy (*p* = 0.074).

**Figure 2:**
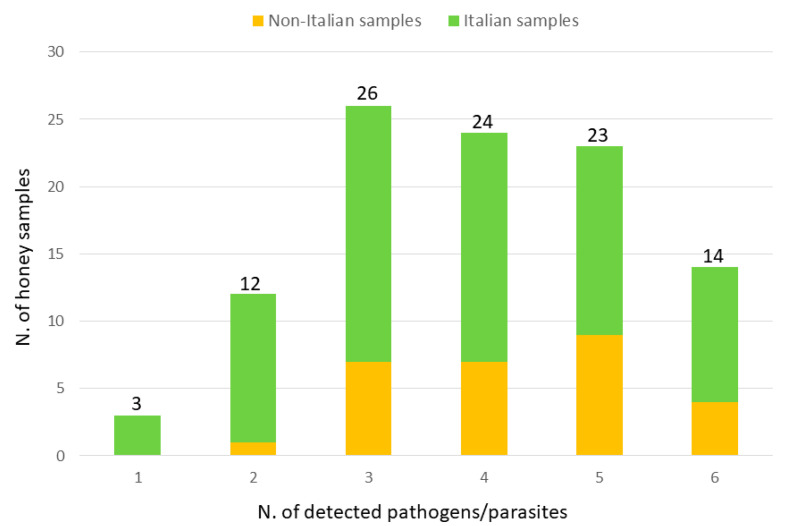
Number of honey samples that were positive for one to six pathogens/parasites differentiated between Italian samples (in green) and non-Italian samples (in orange). The total number of positive samples is reported at the top of each bar.

A total of 14 honey samples (10 of which from Italy) were positive for all pathogens and parasites (excluding those that were not detected in any samples). Both *P. larvae* and *M. plutonius* were co-amplified in 50% of all samples. *Melissococcus plutonius* was always the pathogen that occurred with higher frequency together with all other pathogens or parasites, as expected from its general higher frequency of positive samples. High frequency of co-amplification was observed for *L. passim vs. N. ceranae* (80%; that means that 80% of the samples that were positive for *L. passim* were also positive for *N. ceranae*), for *A. apis vs. N. ceranae* (73%) and for *N. ceranae vs. L. passim* (70%). In these three cases, the co-occurrence of these pairs was statistically different from what would be expected considering the random distribution of each pathogen based on their general frequency of positive samples (*p* < 0.001, *p* < 0.01, and *p* < 0.10, respectively). Only three honey samples (all from Italy) did not show any amplification from all pathogens and parasites (except for *V. destructor*, that was detected in all samples). The distribution of the honey samples that gave amplified fragments for one to six pathogens/parasites is reported in Figure 2.

On average, the Italian samples were positive for 3.78 pathogens/parasites (standard deviation: 1.41) whereas for the non-Italian samples this mean was equal to 4.29 (±2.12). The two means differed at *p* < 0.10.

The two oldest honey samples that were analysed (produced in the year 2004) were positive for *V. destructor*, *P. larvae,* and *M. plutonius*. Considering the year of production, *N. ceranae*, *A. apis,* and *L. passim* were first detected in an Italian honey produced in the year 2012. Due to the unequal distribution of the year of production and the limited number of old samples among those that were analysed (only eight samples were produced in 2013 or earlier), it was not possible to test if there were any differences in the number of positive samples over the years.

## 4. Discussion

Honey is a complex matrix that is mainly made by sugars but that contains several other components that can be explored to disclose interesting information that could be used for several purposes. One of the components, usually neglected or not considered at all, is the DNA that derives from all organisms that directly or indirectly contributed to its productions or that were part of the production systems or environments where the honey has been produced [6,7,43,44,45]. This eDNA also contains information on pathogens and parasites that can be present in the beehive ecological niche and that can represent a threat for the honey bee health.

In this study, we used qualitative PCR-based assays that targeted several pathogens and parasites to identify their presence in honey samples that were produced in Italy and in other countries from all continents. This work should be considered a pilot study that wanted to set up a methodology and evaluate the potential strengths and limits that the use of honey extracted DNA could have for the detection and monitoring of honey bee pathogens and parasites.

DNA from the honey bees is always contained in the honey they produce [44]. Therefore, to evaluate the quality of the extracted DNA and the possibility to use it for other tests, we first amplified the extracted nucleic acid with primers that targeted a region of the honey bee mtDNA that can be also used to detect the mitochondrial lineage of the *A. mellifera* that produced the honey [44]. As all samples gave the expected amplified products for this *A. mellifera* DNA region, the presence or absence of amplification for all other tested primer pairs that targeted nine pathogens or parasites is indicative of the presence or absence of their DNA in the investigated honey samples. DNA could be extracted and then amplified independently by the year of production. DNA from honey produced more than 15 years ago was successfully amplified. Therefore, honey can be used for retrospective analyses and can be stored for this purpose.

All tested assays are qualitative, and in this first pilot study, we did not evaluate the limit of detection for these pathogens that, however, have been already tested in some of the studies from which we derived the primer pairs that we used to amplify honey DNA. It will be interesting to transform the qualitative assays in quantitative analyses that could provide a more precise information and better evaluate the limit of detection from this source of DNA.

It is also worth to mention that the honey samples that we analysed might derive from more than one colony or beehive and probably from more than one apiary. Commercial honey is prepared by mixing the honey derived by more than one beehive. Therefore, the information that we obtained should be referred to the presence of the pathogens and parasites at a higher level than that is usually considered in epidemiological or monitoring studies that usually use the single beehive/colony as the unit of their analyses. The use of honey (particularly, when it does not derive from a single colony, as in our case) might provide information based on the health situation in many colonies or at the apiary level or even at the regional level, depending on the way in which the honey is finally packaged and commercialized. This is an advantage for global monitoring purposes but it could not be considered useful for the punctual health evaluation of a single colony or beehive, that instead could be obtained by traditional approaches or, eventually, by analysing DNA of a single honeycomb (even if this later could not be considered practical in most cases).

The presence or absence of *Paenibacillus larvae*, *Melissococcus plutonius*, *Acarapis woodi,* and *Varroa destructor* in the 102 analysed honey samples was verified using two primer pairs for each targeted organism, obtaining always or for most samples the same results. Some differences in the results of the two assays were observed for the detection of *P. larvae* and *M. plutonius*, probably due to differences in the limits of detection of these tests or for the presence of different strains across samples, having mutations in the primer regions that could affect the amplification efficiency. It will be interesting to further explore this issue not only for these two pathogens but also for the other pathogens and parasites that were amplified in this study by using only one primer pair.

As expected from the global diffusion of *V. destructor*, the DNA of this mite was amplified from all honey samples. The positive amplification obtained for all samples can confirm the high quality of the extracted DNA, as already derived by the successful amplification of the honey bee DNA. We already demonstrated that the sequence information derived by the amplified Varroa DNA fragments from honey samples and obtained using next generation sequencing can provide additional details on the presence of several mite strains in different countries [45]. This approach can be also applied for all other pathogens and parasites investigated in this study if the amplified regions are informative, i.e., they are expected to include sequence differences among strains or lines that could add other information to the derived eDNA.

For two other arthropod parasites, *A. woodi* and *Tropilaelaps* spp., and one microsporidium, *Nosema apis*, we did not identify any positive honey samples. The negative results obtained in these cases could be due to the failure of amplification of the tested primer pairs, even if for two organisms (*A. woodi* and *N. apis*) we used primers that have been already extensively tested by other authors [48,49,50], two primer pairs were tested for *A. woodi* [49,50] with the same negative results, and for all used primers for these three organisms, several PCR conditions were tested (Table 1).

The negative results for *A. woodi* are unexpected, according to the supposed world-wide distribution of this parasite reported by a few studies [35,50,53,54]. However, other studies indicated that the prevalence and diffusion of this honey bee tracheal mite is limited and potentially related to local conditions and factors [55,56,57] and this picture might better match the results we obtained. Other studies are needed to confirm the negative results derived by our approach based of the amplification of honey DNA by two different PCR assays that have been specifically designed to detect this species [49,50].

On the other hand, the negative results for *Tropilaelaps* spp. were to some extent expected. That means that the absence of *Tropilaelaps* spp. amplified fragments indicates that these damaging mites [40] did not spread into the regions where we sampled the analysed honey. It could be also possible that the prevalence of mites of this genus was not relevant in these areas or it was under the detection limit of this analysis. It is also worth to mention that the assay we developed should be further tested using some positive honey samples. To be sure that honey DNA can be useful to capture the presence of these emerging parasites, it will be important to develop other PCR assays able to confirm these results. Anyway, even if the absence of *Tropilaelaps* spp. amplification is a good news, it further raises the attention to the usefulness of monitoring methods, like what we propose in this study, to prevent potential spreads in Europe or America where these mites would be probably very damaging to the apiculture sector [41].

Another pathogen that was never detected in the analysed samples was *N. apis*. The relevance of this microsporidium has been decreasing over the last decades that evidenced a corresponding increase of the infection prevalence and diffusion of *N. ceranae,* which has been suggested to replace *N. apis* [27,58,59,60]. Our results might confirm this general trend. *Nosema ceranae* was observed in more than 50% of the analysed samples further demonstrating the widespread and general prevalence of this obligate intracellular eukaryotic parasite.

*Lotmaria passim*, currently considered the predominant trypanosomatid species in *A. mellifera* host populations [33], was detected in about 50% of the honey samples, 80% of which were also positive for *N. ceranae* (and this co-occurrence was not derived by chance), confirming, to some extent, that its presence is usually associated with this microsporidium [34,61]. It is of course clear that, just by analysing honey DNA, is it not possible to establish a direct relationship between these two infection agents but this speculative deduction could open new opportunities to interpret the results when larger number of samples and specimens purposely collected for co-occurrence evaluations are investigated. It was also interesting to note that, stratifying the results of the Italian samples based on the two main geographic areas from which the largest number of samples were collected, *L. passim* occurred more frequently in the honey produced in the North of Italy than in that produced in the South of Italy. It will be important to increase the number of samples to confirm this preliminary evidence, that at present it does not have any clear explanations.

*Ascosphaera apis*, the agent of chalkbrood, was detected in more than 40% of all samples, confirming also for this entomopathogenic fungus the widespread distribution already known [29]. It will be important to understand if the DNA of *A. apis* derives from spores (which can have up to 15 years viability; [29]) or other materials, for the potential obvious implications derived by the possibility to further spread this disease through the exchange of honey. Similarly to what was discussed for *L. passim*, this fungus was detected in honey samples that in 73% of cases were also positive for *N. ceranae*, further suggesting a possible relationship determining the co-occurrence of these pathogens (of course taking into account what already discussed above).

*Melissococcus plutonius* was the most frequent pathogen amplified in the 102 honey samples, with 87% of positive samples. This Gram-positive lanceolate coccus is the agent of the European foulbrood, one of the most serious brood diseases of *A. mellifera*, for which there is still a poor understanding of disease epidemiology. It is already known that this bacterium is also present in healthy colonies, variants of this agent have been described and that the disease can occur in combination with other health threats [62,63,64]. Anyway, the very high frequency of positive samples is quite puzzling and needs to be further investigated to better understand its meaning, by taking into account the methodology that was used for this detection. The lower frequency of positive samples produced in the North of Italy than those produced in the South of Italy is also another matter of further evaluation, if confirmed by analysing a larger number of samples.

The general quite high percentage of positive honey samples for *P. larvae* indicates that not in all cases the presence of this pathogen might cause American foulbrood, as already reported [65,66]. The results of the amplifications for the agent of this disease showed that this bacterium was less frequent in the Italian samples (49%) than in the non-Italian samples (79%) and this difference was highly significant. The interpretation of this result is not simple and might be derived by different management systems and practices or environmental conditions that would limit the diffusion of this Gram-positive pathogenic bacterium in Italy compared to other parts of the world. This is in line to the lower mean number of pathogens/parasites detected in the Italian samples than in the non-Italian samples. These first results might speculatively indicate general better healthy conditions of the colonies in Italy than in other countries—a hypothesis that should be supported by other evidences. Another explanation for this difference could be due to the fact that non-Italian samples might be derived by a larger number of beehives or apiaries (most of them were purchased and not directly provided by the beekeepers; Table S1) than the Italian samples (most of which were provided directly by the beekeepers; Table S1), increasing the possibility to include honey derived by positive beehives or beehives in which this pathogen was present. If this would be a possible explanation of this result, it is not clear, however, why this difference between Italian and non-Italian honey samples was observed only for *P. larvae* and not also for other pathogens.

## 5. Conclusions

This study reported for the first time an extensive use of honey eDNA to design epidemiological and monitoring approaches for pathogens and parasites with the final objective of obtaining a general picture of the sanitary status of the honey bee populations at macro-levels (i.e., apiary, beekeeper, regions, countries, continents). Using this unconventional approach, we also obtained, for the first time, a comprehensive analysis (even if preliminary) of the distribution and frequency of several pathogens and parasites in Italy. It will be useful (i) to refine and improve the applied assays, adding methods to detect other pathogens, (ii) to increase the number of the analysed honey samples to improve the interpretation of the results, and (iii) to correlate the results derived by the DNA analyses with the situations in the colony and/or apiary and epidemiological data and distribution of pathogens and parasites in a region that could be obtained from direct observations. The results of this study on the distribution, co-occurrence and prevalence of some of the targeted pathogens and parasites should be interpreted, considering that honey bee health threats cannot be regarded as local problems.

## Figures and Tables

**Table 1:** Information on PCR primers and PCR conditions used in this study to amplify DNA extracted from the 102 honey samples reported in Table S1.

Species	Primer Name ^1^	Primer Sequences (5’-3’): Forward and Reverse ^2^	Size in Bp	Amplified Region	Ta °C ^3^	Reference
*Apis mellifera*	AM_Forward AM_Reverse	GGCAGAATAAGTGCATTG TTAATATGAATTAAGTGGGG	C 85, M 139, A 153 ^4^	mtDNA COI-COII	51	[44]
*Paenibacillus larvae*	AF_6f AF_7r	GCAAGTCGAGCGGACCTTGT TCGTCGCCTTGGTAAGC	237	16S rRNA	63	[14]
*Paenibacillus larvae*	Han233PaeLarv16S_F Han233PaeLarv16S_R	GTGTTTCCTTCGGGAGACG CTCTAGGTCGGCTACGCATC	233	16S rRNA	59	[46]
*Melissococcus plutonius*	MeliFORa MeliREVa	GTTAAAAGGCGCTTTCGGGT GAGGAAAACAGTTACTCTTTCCCCTA	281	16S rRNA	63	[36]
*Melissococcus plutonius*	Mp_Arai187_F Mp_Arai187_R	TGGTAGCTTAGGCGGAAAAC TGGAGCGATTAGAGTCGTTAGA	187	NapA	59	[47]
*Nosema apis*	Nose_apis_chen_F Nose_apis_chen_R	CCATTGCCGGATAAGAGAGT CCACCAAAAACTCCCAAGAG	269	SSUrRNA	54–60 ^5^	[48]
*Nosema ceranae*	Nose_cera_chen_F Nose_cera_chen_R	CGGATAAAAGAGTCCGTTACC TGAGCAGGGTTCTAGGGAT	250	SSUrRNA	58	[48]
*Lotmaria passim*	LpCytb_F1 LpCytb_R1	CGAAGTGCACATATATGCTTTAC GCCAAACACCAATAACTGGTACT	247	mtDNA cyt b	59	[34]
*Ascosphaera apis*	AscosFORa AscosREVa	TGTGTCTGTGCGGCTAGGTG GCTAGCCAGGGGGGAACTAA	136	18S rRNA	61	[36]
*Acarapis woodi*	Acarap_cox1_F1 Acarap_cox1_R1	CAGTAGGGCTAGATATCGATACCCGAGCTT TGAGCTACAACATAATATCTGTCATGAAGA	247	mtDNA Cox 1	55–62 ^5^	[49]
*Acarapis woodi*	Acarapis_cox1_F2 Acarapis_cox1_R2	CGGGCCCGAGCTTATTTTACTGCTG GCGCCTGTCAATCCACCTACAGAAA	162	mtDNA Cox 1	55–62 ^5^	[50]
*Varroa destructor*	Varr_cox1_181_F Varr_cox1_181_R	GCCTTTATTTGTATGGTCTG TGGGTGTCCAAAAAATCA	181	mtDNA Cox 1	52	[45]
*Varroa destructor*	Varr_cox1_148_F Varr_cox1_148_R	TGGTATTATTTCTCATGTAATTTG ATCAATATCTATTCCTACTGTAAA	148	mtDNA Cox 1	55	[45]
*Tropilaelaps clareae* ^6^	Tropi_cox1_F Tropi_cox1_R	TATTTGTATGATCTGTCCTA ATAATACCAAATCCTGGTA	214	mtDNA Cox 1	54–60 ^5^	This study

^1^ Internal name of the forward and reverse primers. ^2^ Sequence of the forward and reverse primers. ^3^ Annealing temperature used in the PCR amplifications (°C). ^4^ The amplified fragment can have different size according to the mitochondrial lineage (A, C or M) as described in Utzeri et al. [44]. ^5^ Range of the tested annealing temperature. ^6^ The PCR assay was designed on the mitochondrial DNA sequence of *Tropilaelaps clareae* (GenBank accession no. EF025461). However, it is not specific for this species, as according to the sequence alignment with the corresponding region of other *Tropilaelaps* species, these primers can also amplify other species of this genus (i.e., *T. mercedesae*, *T. thaii* and *T. koenigerum*) due to 100% identity of their mtDNA sequences in the primer regions. For this reason, the assay was indicated to amplify DNA of *Tropilaelaps* spp. The primer pair does not amplify *V. destructor* DNA.

## Supplementary Materials

The following are available online at https://www.mdpi.com/2306-7381/7/3/113/s1, Figure S1: Distribution of the analysed honey samples based on the years of production. Table S1: List and details of the investigated honey samples.
