# Supplementary material for: Honey as a Source of Environmental DNA for the Detection and Monitoring of Honey Bee Pathogens and Parasites
Source: Vet Sci. 2020 Aug 15;7(3):113. doi: 10.3390/vetsci7030113 (PMC7558659; doi:10.3390/vetsci7030113)

Table S1. List and details of the investigated honey samples.

Laboratory number	Honey sample[1]	Continent	Country	Region	Province/Town	Year	Type/Origin
1	Eucalyptus Honey	Europe	Italy	Veneto	Vicenza	2016	Monofloral
2	Apple Tree Honey	Europe	Italy	Trentino-Alto Adige	Trento	2015	Monofloral
3	Cistus/Rock rose Honey	Europe	Italy	Sardinia	Oristano	2015	Monofloral
4	Valdivia Honey	America	Chile	Valdivia	N/A	2014	Polyfloral
7	Thistle Honey	Europe	Italy	Sardinia	Oristano	2014	Monofloral
8	Chestnut Honey	Europe	Italy	Piedmont	Novara	2015	Monofloral
9	Linden/Lime Tree Honey	Europe	Italy	Friuli-Venezia Giulia	Udine	2015	Monofloral
10	Lavender Honey	Europe	Italy	Sardinia	Sassari	2015	Monofloral
11	Sunflower Honey	Europe	Italy	Toscana	Pistoia	2015	Monofloral
12	Dandelion Honey	Europe	Italy	Emilia-Romagna	Modena	2013	Monofloral
13	Acacia Honey	Europe	Italy	Toscana	Arezzo	2015	Monofloral
14	Coriander Honey	Europe	Italy	Umbria	Gubbio	2015	Monofloral
16	Cherry Tree Honey	Europe	Italy	Sardinia	Oristano	2016	Monofloral
17	Alfa-Alfa Honey	Europe	Italy	Emilia-Romagna	Ravenna	2016	Monofloral
18	Forest Honeydew Honey	Europe	Italy	Emilia-Romagna	Modena	2016	Honeydew
19	Honeydew Honey	Europe	Italy	Lombardia	Bergamo	2016	Honeydew
20	Tree of Heaven Honey	Europe	Italy	Emilia-Romagna	Bologna	2016	Monofloral
21	Manuka Honey*	Oceania	New Zealand	N/A	N/A	2016	Monofloral
22	Artichoke Honey	Europe	Italy	Sicily	Palermo	2015	Monofloral
23	Asphodel Honey	Europe	Italy	Sardinia	Cagliari	2014	Monofloral
24	Linden/Lime Tree Honey	Europe	Italy	Sardinia	South Sardinia	2015	Monofloral
25	Silver Fir Honeydew Honey	Europe	Italy	Piedmont	Cuneo	2017	Honeydew
26	Forest Honeydew Honey	Europe	Italy	Veneto	Verona	2016	Honeydew
27	Forest Honeydew Honey	Europe	Italy	Trentino-Alto Adige	Bolzano	2017	Honeydew
28	Quercus Honeydew Honey	Europe	Italy	Veneto	Verona	2018	Honeydew
29	Silver Fir Honeydew Honey	Europe	Italy	Emilia-Romagna	Forlì-Cesena	2014	Honeydew
30	Polyfloral Honey	Europe	Italy	Sicily	Palermo	2016	Polyfloral
31	Spruce Honeydew Honey	Europe	Italy	Valle D'Aosta	Aosta	2017	Honeydew
32	Onion Honey	Europe	Italy	Abruzzo	Chieti	2018	Monofloral
33	Bramble Honey	Europe	Italy	Abruzzo	Chieti	2018	Monofloral
34	Hawthorn/Whitethorn Honey	Europe	Italy	Abruzzo	Chieti	2017	Monofloral
35	Dill Honey	Europe	Italy	Sicily	Palermo	2017	Monofloral
36	Prickly pear Honey	Europe	Italy	Sicily	Catania	2017	Monofloral
37	Ferula Honey	Europe	Italy	Sicily	Filicudi Island	2017	Monofloral
38	Eucalyptus Honey	Europe	Italy	Calabria	N/A	2017	Monofloral
39	Thyme Honey	Europe	Italy	Sardinia	South Sardinia	2017	Monofloral
40	Sulla Honey	Europe	Italy	Campania	Salerno	2014	Monofloral
41	Orange Tree Honey	Europe	Italy	Sicily	Caltanissetta	2014	Monofloral
42	Rosemary Honey	Europe	Italy	Sardinia	South Sardinia	2017	Monofloral

43	Beach Flower Honey	Europe	Italy	Sicily	Lampedusa Island	2017	Polyfloral
44	Polyfloral Honey	Europe	Croatia	Dalmatia	Gizdavac	2015	Polyfloral
45	Polyfloral Honey	Europe	Croatia	Bjelovar	Daruvar	2015	Polyfloral
46	Polyfloral Honey	Asia	Japan	Honshu Island	Kyoto	2016	Polyfloral
47	Polyfloral Honey	America	Brazil	Rio Grande do Sul	Cachoeirinha	2015	Polyfloral
48	Polyfloral Honey	Europe	Croatia	Croatia proper	Zagreb	2015	Polyfloral
49	Polyfloral Honey	America	USA	California	Ukiah	2016	Polyfloral
50	Polyfloral Honey	America	USA	Colorado	Greeley	2016	Polyfloral
51	Acacia Honey	Europe	Holland	Gerderland	Beesd	2017	Monofloral
52	Polyfloral Honey	Asia	China	N/A	N/A	2017	Polyfloral
53	Lavender Honey	Europe	France	Provence	N/A	2017	Monofloral
54	Polyfloral Honey	Asia	China	Dongbei	N/A	2017	Polyfloral
55	Polyfloral Honey	Asia	China	N/A	N/A	2017	Polyfloral
56	Polyfloral Honey	America	Guatemala	Petén	Santa Elena	2017	Polyfloral
57	Polyfloral Honey	Europe	Serbia	N/A	N/A	2017	Polyfloral
58	Thyme Honey	Europe	Greece	Lefkada Island	Dragano	2012	Monofloral
59	Polyfloral Honey	America	Brazil	N/A	N/A	2016	Polyfloral
60	Polyfloral Honey	Asia	India	N/A	N/A	2016	Polyfloral
61	Polyfloral Honey	America	USA	Iowa	Scranton	2016	Polyfloral
62	Polyfloral Honey	Africa	Ethiopia	N/A	N/A	2016	Polyfloral
64	Polyfloral Honey	Europe	Italy	Basilicata	Potenza	2017	Polyfloral
65	Citrus Fruits Honey 1	Europe	Italy	Calabria	Reggio Calabria	2017	Monofloral
66	Limonium Honey	Europe	Italy	Veneto	Venezia	2017	Monofloral
67	Citrus Fruits Honey 2	Europe	Italy	Calabria	Cosenza	2017	Monofloral
68	Acacia Honey	Europe	Italy	Calabria	Catanzaro	2017	Monofloral
69	Spruce Honeydew Honey	Europe	Italy	Veneto	Belluno	2017	Honeydew
70	Honeydew Honey	Europe	Italy	Valle D'Aosta	Aosta	2017	Honeydew
71	Ferula Honey	Europe	Italy	Sardinia	South Sardinia	2017	Monofloral
72	Polyfloral Honey	Europe	Italy	Sardinia	South Sardinia	2017	Polyfloral
73	Thistle Honey	Europe	Italy	Sardinia	Oristano	2017	Monofloral
74	Tangerine Honey	Europe	Italy	Sicily	Catania	2018	Monofloral
75	Sulla Honey	Europe	Italy	Molise	Campobasso	2017	Monofloral
76	Acer Honey	Europe	Italy	Friuli-Venezia Giulia	Udine	2017	Monofloral
77	Linden Tree Honey	Europe	Italy	Friuli-Venezia Giulia	Trieste	2018	Monofloral
78	Marasca Cherry Honey	Europe	Italy	Friuli-Venezia Giulia	Trieste	2018	Monofloral
82	Acacia Honey	Europe	Hungary	N/A	N/A	2017	Monofloral
83	Avocado Honey	America	Mexico	N/A	N/A	2016	Monofloral
84	Forest Honey	Europe	Finland	Tavastia Proper	N/A	2018	Polyfloral
85	Linden Tree Honey	Europe	Finland	Uusimaa	Espoo	2018	Monofloral
86	Wood Forest Honey	Europe	Finland	Kanta-Hame	Tammela	2018	Polyfloral
87	Buckwheat Honey	Europe	Finland	Uusimaa	Tuusula	2018	Monofloral
88	Lemon Tree Honey	Europe	Italy	Sicily	Catania	2018	Monofloral
90	Polyfloral Honey	Europe	Italy	Emilia-Romagna	Ravenna	2018	Polyfloral

91	Sulla Honey	Europe	Italy	Sicily	N/A	2013	Monofloral
92	Chestnut Honey	Europe	France	Corsica	N/A	2015	Monofloral
93	Polyfloral Honey	Europe	Italy	Emilia-Romagna	Ravenna	2004	Polyfloral
94	Linden Tree Honey	Europe	Italy	N/A	N/A	N/A	Monofloral
95	Polyfloral Honey	Europe	Italy	Emilia-Romagna	Ravenna	N/A	Polyfloral
96	Polyfloral Honey	Europe	Italy	Emilia-Romagna	Ravenna	2007	Polyfloral
97	Chestnut Honey	Europe	Italy	Piedmont	Cuneo	2012	Monofloral
98	Polyfloral Honey	Europe	Italy	Emilia-Romagna	Reggio Emilia	2010	Polyfloral
99	Polyfloral Honey	Europe	Italy	N/A	N/A	N/A	Polyfloral
100	Citrus Fruit Honey	Europe	Italy	Emilia-Romagna	Ravenna	2004	Polyfloral
101	Heaven Tree Honey	Europe	Italy	Lazio	Roma	N/A	Monofloral
102	Acacia Honey	Europe	Italy	Lazio	Frosinone	N/A	Monofloral
103	Ziziphus Honey	Europe	Italy	Lazio	Viterbo	N/A	Monofloral
104	Acacia Honey	Europe	Italy	Liguria	La Spezia	N/A	Monofloral
105	Acacia Honey	Europe	Italy	Liguria	Genova	N/A	Monofloral
106	Polyfloral Honey	Europe	Italy	Marche	Fermo	N/A	Polyfloral
107	Sunflower Honey	Europe	Italy	Marche	Ancona	N/A	Monofloral
108	Polyfloral Honey	Europe	Italy	Puglia	Brindisi	N/A	Polyfloral
109	Cherry Tree Honey	Europe	Italy	Puglia	Bari	N/A	Monofloral
110	Rosemary Honey	Europe	Italy	Puglia	Foggia	N/A	Monofloral

N/A = not available.[1] Samples marked with an asterisk ("*") were purchased. All other samples were provided by the beekeepers.

Figure S1. Distribution of the analysed honey samples based on the years of production. N/A indicates that this information was not available.

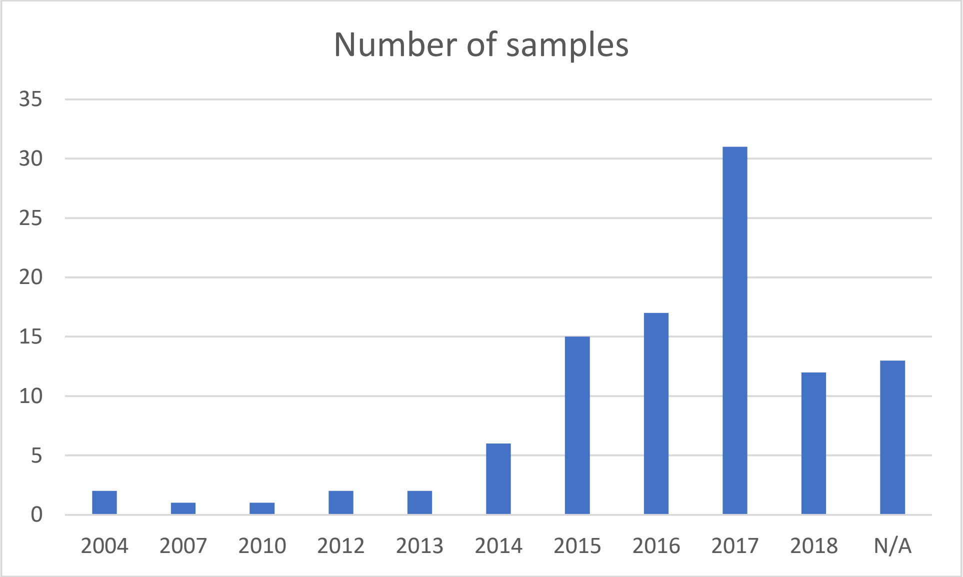

Supplement: Supplementary file 1 [file vetsci-07-00113-s001.pdf]
